# Inhibition or Facilitation? Contrasted Inter-Specific Interactions in *Sphagnum* under Laboratory and Field Conditions

**DOI:** 10.3390/plants9111554

**Published:** 2020-11-12

**Authors:** Chao Liu, Zhao-Jun Bu, Azim Mallik, Yong-Da Chen, Xue-Feng Hu, Fan Lu

**Affiliations:** 1 Key Laboratory of Geographical Processes and Ecological Security in Changbai Mountains, Ministry of Education, School of Geographical Sciences, Northeast Normal University, Renmin 5268, Changchun 130024, China; liuc272@nenu.edu.cn (C.L.); chenyd880@nenu.edu.cn (Y.-D.C.); huxf@cib.ac.cn (X.-F.H.); luf785@nenu.edu.cn (F.L.); 2 State Environmental Protection Key Laboratory of Wetland Ecology and Vegetation Restoration, Institute for Peat and Mire Research, Northeast Normal University, Renmin 5268, Changchun 130024, China; 3 Jilin Provincial Key Laboratory for Wetland Ecological Processes and Environmental Change in the Changbai Mountains, Renmin 5268, Changchun 130024, China; 4 Department of Biology, Lakehead University, Thunder Bay, ON P7B 5E1, Canada; amallik@lakeheadu.ca; 5 University of Chinese Academy of Sciences, No. 19A Yuquan Road, Beijing 100049, China

**Keywords:** *Sphagnum*, phenolics, phenotypic responses, inter-specific interactions

## Abstract

In a natural environment, plants usually interact with their neighbors predominantly through resource competition, allelopathy, and facilitation. The occurrence of the positive effect of allelopathy between peat mosses (*Sphagnum* L.) is rare, but it has been observed in a field experiment. It is unclear whether the stability of the water table level in peat induces positive vs. negative effects of allelopathy and how that is related to phenolic allelochemical production in *Sphagnum*. Based on field experiment data, we established a laboratory experiment with three neighborhood treatments to measure inter-specific interactions between *Sphagnum angustifolium* (Russ.) C. Jens and *Sphagnum magellanicum* Brid. We found that the two species were strongly suppressed by the allelopathic effects of each other. *S. magellanicum* allelopathically facilitated *S. angustifolium* in the field but inhibited it in the laboratory, and relative allelopathy intensity appeared to be positively related to the content of released phenolics. We conclude that the interaction type and intensity between plants are dependent on environmental conditions. The concentration of phenolics alone may not explain the type and relative intensity of allelopathy. Carefully designed combined field and laboratory experiments are necessary to reveal the mechanism of species interactions in natural communities.

## 1. Introduction

Allelopathy and resource competition are known to be two important mechanisms of species interaction that often work together [1]. Allelopathy is referred to as a form of interference competition mediated by allelochemicals released into the environment [2]. Both allelopathy and competition can drive basic ecosystem processes and determine ecosystem functions [1]. As primitive plants, bryophytes have evolved to possess allelopathy and resource competition to coexist with vascular plants [3]. For example, *Sphagnum* L. could release secondary metabolites that not only suppress litter decomposition [4,5] but also exhibit strong allelopathic effects that reduce the growth of vascular plants [6,7] and affect microbial community composition [8,9] while indirectly influencing the carbon stock in peatlands [10]. Additionally, *Sphagnum* could achieve its resource competition by producing more side-shoots than its neighbors—side-shoot production could reflect clonal expansion [11]; more productive side shoots can overtop and shade its neighbors [12] and gain competitive advantages. Since *Sphagnum* is the dominant genus in northern peatlands and contributes up to 50% of aboveground production [13], attention to the plant–plant interactions of co-occurring *Sphagnum* species is critical to understand their community structure in peatlands.

Recently, several studies have focused on the role of allelopathy in plant–plant interactions among *Sphagnum* species [14,15]. Studies on the allelopathy of *Sphagnum* have mainly estimated the effects of their aqueous extracts or exudates on the growth of other plants of the same genus, and these studies may have underestimated (e.g., Ingerpuu and Vellak (2013)) or exaggerated (e.g., Michel et al., 2011) [14,16] its allelopathic effect. Because such experiments are designed to maximize the release of allelochemicals, these effects may be induced by other factors (extract pH, osmotic potential, and high concentrations of mineral elements and other organic molecules in cell fluid) than just allelopathy [1]. Previous studies have collected the exudates of plants grown in a monoculture, ignoring the fact that additional allelochemicals may be produced in the presence of neighbors. A more realistic method of determining allelopathy might be achieved by exposing plants to allelochemicals from healthy neighbors (i.e., grown with neighbors).

Plant–plant interactions may also vary with environmental conditions that may shift competition to facilitation with increasing environmental stress, as per the stress gradient hypothesis [17]. In the field, facilitation among bryophytes was observed in a non-rainy season [18]. However, under laboratory conditions with continuous moisture, a positive interaction (both resource competition and allelopathy) between the two species may not found. In a drought treatment, an increase in the species richness of bryophyte communities led to a biomass increase, while this relationship was not found in a moist environment [19]. Økland (1996) suggested that facilitation among bryophytes is related to inter-specific difference in water conservation [20].

Allelopathy is generally exhibited as a mechanism of inhibition [1,17]; relatively few studies have reported its stimulatory effects [2]. Qin et al. (2018) observed that a low concentration of aqueous extracts of *Eucalyptus urophylla* produced positive effects on the growth of *Schefflera octophylla*, *Cinnamomum camphora*, and *Helicia cochinchinensis* [21]. In a previous field experiment, Liu et al. (2020) reported that *Sphagnum magellanicum* Brid. promoted the growth of *Sphagnum angustifolium* (Russ.) C. Jens by allelopathy in hollow habitats [22]. These authors proposed that such a positive effect may be attributed to the dilution effect of allelochemicals in peat water because the positive effects of allelopathy result at a low allelochemical content and negative effects result at a high allelochemical content [23]. Facilitation among bryophytes might be the net result of the negative and positive effects of allelochemicals mediated by content and concentration.

Field experiments could reflect the complex reality of many interacting factors that are hard to control and standardize that may obscure or reduce the contribution of inter-specific interactions [24]. Laboratory experiments can assess the role of independent variables and exaggerate the contribution of inter-specific interactions [25]. Hence, to better reveal the role of inter-specific interactions, especially the positive effect of allelopathy in plant coexistence, we conducted a laboratory experiment to make a comparison with a previous field experiment (Liu et al. 2020) using the same two *Sphagnum* species; *S. angustifolium*, a hollow inhabiting species, and *S. magellanicum,* a hummock species. Since negative inter-specific interactions predominate in a benign environment [26], we hypothesized that: (1) a negative effect of allelopathy would be observed in the laboratory where water table level was stable, and (2) the phenolic production of *Sphagnum* is negatively related to relative allelopathy intensity, showing inhibition at a high phenolics content and facilitation at a low phenolics content.

## 2. Materials and Methods

### 2.1. Study Site

Dongfanghong peatland (42°11′ N, 128°19′ E) of the Changbai Mountains, Northeastern China was chosen for *Sphagnum* collection, and it was also the field experiment site in a former study [22]. Here, the annual average temperature is 2.2 °C, and the annual precipitation is 780 mm (concentrated in June–August). The peatland is large (c. 170 km^2^), with diverse vegetation and ecohydrological conditions. The stand where the field experiment was set up is characterized as a poor fen with pH 5.1. The vegetation is dominated by *Sphagnum fuscum* (Schimp.) Klinggr., *S. magellanicum* and *S. angustifolium*, including some tree species such as *Larix olgensis* A. and *Betula fruticosa* var. *ruprechtiana* Trautv. and *Carex* spp.

### 2.2. Experimental Design

In September 2016, shoots of *Sphagnum* were cut to 9.0 cm-long and then inserted into PVC (polyvinyl chloride) boxes at a natural density (100 and 80 shoots/100 cm^2^ for *S. angustifolium* and *S. magellanicum*, respectively). The experimental design involved two species and three inter-specific interactions: (1) a monoculture of *S. angustifolium* and *S. magellanicum* growing separately (hereafter referred to as Mono). (2) a mixed culture without AC (referred to as Mix), and (3) a mixed culture with AC added to the neighbor (referred to as Mix_AC_). In total, there were 6 treatments and 5 replicates for each treatment. Activated charcoal can remove inhibitory compounds, including phenolics, released by plants [27,28,29,30], which can largely reduce or even remove the allelopathic effect of *Sphagnum*. We added 1.5 g (312.5 g m^-2^) of AC (Sigma-Aldrich, St. Louis, MO, USA, untreated, granular, 3 × 5 mm) to 0.5 cm below the donor species.

In this study, the inter-specific interaction was measured as the total interaction, resource competition, and allelopathy. The methods for assessing these effects were as follows. (1) The effects of the total inter-specific interaction of donor *Sphagnum* on target *Sphagnum* were assessed by comparing their traits (biomass production, side-shoot production. and soluble sugar content) in the monoculture and the mixed culture with AC; (2) the effects of resource competition of donor *Sphagnum* on target *Sphagnum* were assessed by comparing their traits in the monoculture and mixed culture with AC; and (3) the effects of the allelopathy of donor *Sphagnum* on target *Sphagnum* were assessed by comparing their traits in the AC-free culture and mixed culture with AC added to the neighbor.

The experimental design in the laboratory was consistent with the field experiment, except for the water table level. In the field, two water table levels—low (an average 24 cm below moss surface, hummock habitat) and high (an average 12 cm below moss surface, hollow habitat)—were used [22]. A positive effect of allelopathy was observed only in the hollow habitat, and, hence, for comparison in similar conditions, we only used a water table level similar to that in the hollow habitats in our laboratory experiment. Specifically, the *Sphagnum* shoots were cultured in a growth chamber (HPG-400HX, Harbin Donglian Electronic and Technology Development Co. Ltd., Harbin, China). To simulate the climate of Dongfanghong peatland during the middle growing season, the temperature, air humidity, and duration in day and night were set to 22 and 18 °C, 70 and 90%, and 16 and 8 h, respectively [31] The water level in the boxes was kept at 3 cm, which made the humidity of growth chamber close to wet conditions, similar to hollow habitats in the field. Every second day, 4 mL of distilled water was sprayed onto the capitula of *Sphagnum* shoots [31]. Every week, 6.0 mL of Rudolph’s nutrient solution was added to each box [32]. The position of each box was randomized in the growth chamber.

### 2.3. Growth and Biochemical Trait Measurement

The field experiment lasted for one year with a growing season for approximately 180 d. The laboratory experiment lasted for 10 weeks. At the end of both experiments, the shoot bundles in each sample were taken out, and the number of side-shoots (side-shoot production) was counted. To determine biomass production, the part between the capitula (the top 1 cm part) and the lower 8 cm stem was oven-dried at 70 °C for 24 h and weighed [31]. The remaining part of *Sphagnum* was also dried at 70 °C for 24 h. All oven-dried shoots were ground, and the contents of the reserved phenolics, soluble sugars, starch (referred to as NSC), and cellulose in shoots, as well as the concentration of released phenolics in the leachates, were analyzed. Before measuring the released phenolics, the ground material was dissolved in 20 mL of 40% alcohol and then extracted in a modified microwave (Yuhua WBFY-201, Chengdu Dilaiya Trade Co. Ltd., China) for 90 s. Phenolics were measured spectroscopically at an absorbance of 765 nm using Folin–Ciocalteu with gallic acid as standard [33]. The contents of soluble sugar, starch, and cellulose were determined by the anthrone method [34] using 80% ethanol to extract soluble sugar, 9.2 mol L^−1^ and 4.6 mol L^−1^ HClO_4_ solutions to extract starch, and 60% sulfuric acid to extract cellulose. The standard substances for measuring soluble sugar, starch, and cellulose were glucose, glucose, and pure cellulose, respectively. The reagent anthrone–sulfuric acid was used to spectroscopically measure soluble sugar, starch, and cellulose contents at the 620 nm wavelength. Carbon and nitrogen contents were determined by an element analyzer (Euro Vector, Pavia, Italy). Phosphorus was measured by an automated discrete analyzer (SmartChem 140, AMS-Alliance, Guidonia, Italy).

### 2.4. Data Processing and Statistical Analysis

To test the neighbor effect and interaction type, we used the *RII* index [35]:

*RII _TI_* = (*P _Mix_* − *P _Mono_*)/(*P _Mix_* + *P _Mono_*) (1)

*RII _RC_* = (*P _MixAC_ − P _Mono_*)/(*P_MixAC_ + P _Mono_*) (2)

*RII _A_* = (*P _Mix_ − P _MixAC_*)/(*P _Mix_ + P _MixAC_*) (3)

where *TI*, *RC,* and *A* are the total inter-specific interaction, resource competition, and allelopathy, respectively. Meanwhile, *P _Mono_*, *P _Mix_*, and *P _MixAC_* are the performance of *Sphagnum*, respectively, in the monoculture, the mixed culture without AC, and the mixed culture with AC. A positive value of the *RII* indicated facilitation, and a negative value indicated suppression. The *RII* was calculated for biomass production and side-shoot production.

To determine the plasticity response of *Sphagnum* to its neighbors, we calculated the phenotypic plasticity index (*PI*) of *Sphagnum* [36] as follows:

*PI _TI, RC or A_* = (*Trait _mix_ − Trait _mono_*)/*Trait _MAX_* (4)

where *Trait _mix_* and *Trait _mono_* are the traits of *Sphagnum* in the monoculture and the mixed culture without AC, respectively. *Trait _MAX_* is the maximum between *Trait _mix_* and *Trait _mono_*. For instance, *PI _TI_* was calculated as follows: (trait performance in the mixed culture without AC—trait performance in monoculture)/maximal trait performance between the monoculture or the mixed culture without AC. The phenotypic plasticity index ranged from −1 to + 1. Close to “0” meant that the trait had no response to the neighbor, close to −1 and + 1 meant that the response of the trait to the neighbor was strong. A positive value indicated facilitation, and a negative value indicated suppression. The *PI* was calculated for height increment, side-shoot production, soluble sugar content, starch content, cellulose content, carbon content, nitrogen content, and phosphorus content.

All statistical analyses were performed using SPSS Statistics 19.0 package (SPSS Inc., Chicago, IL, USA). A two-way ANOVA was used to examine the main effect of the culture conditions (laboratory and field) and inter-specific interactions (in the monoculture, the mixed culture without AC, and the mixed culture with AC added to the neighbor) on plant traits (biomass production, side-shoot production, reserved phenolics content, released phenolics content, soluble sugar content, starch content, cellulose content, carbon content, nitrogen content, and phosphorus content) of each species in the laboratory and the field (the data of biomass production, side-shoot production, soluble sugar content, and starch content of *Sphagnum* in the field were from Liu et al. 2020). We used a one sample *t*-test to test whether *RII* or *PI* values significantly differed from a hypothesized mean value = 0. Analytical significance levels were accepted at *p*-value < 0.05.

## 3. Results

### 3.1. Responses of Morphological and Biochemical Traits to Culture Conditions and Neighbors

**Figure 1:**
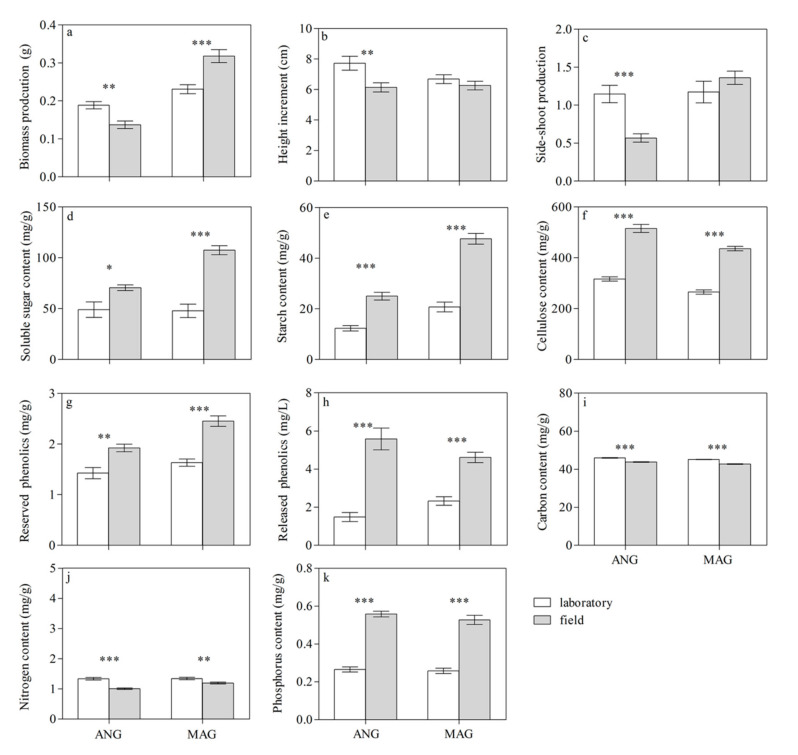
Effect of culture conditions on the morphological traits—(**a**) biomass production, (**b**) height increment, and (**c**) side-shoot production—and biochemical traits—(**d**) soluble sugar content, (**e**) starch content, (**f**) cellulose content, (**g**) reserved phenolics content, (**h**) released phenolics content, (**i**) carbon content, (**j**) nitrogen content, and (**k**) phosphorus content—of *S. angustifolium* (ANG) and *S. magellanicum* (MAG) in a monoculture. Data are mean ± 1SE (Standard Error, *n* = 5). Asterisks represent significant differences in the traits of each species between the laboratory and the field. *: *p* < 0.05; **: *p* < 0.01; and ***: *p* < 0.001. The data of biomass production and side-shoot production, as well as the reserved phenolics, released phenolics, soluble sugar, and starch contents, of *Sphagnum* in the field were from Liu et al. (2020).

As shown in Table 1, culture conditions had clear impacts on the morphological and biochemical traits of both *Sphagnum* species (*p* < 0.05 for all), except for the height increment and side-shoot production in *S. magellanicum*. The neighbor influenced the starch content, cellulose content, and reserved phenolics content of *S. angustifolium* (*p* < 0.01 for all), as well as the side-shoot production, released phenolics concentration, cellulose content, carbon content, nitrogen content, and reserved phenolics content of *S. magellanicum* (*p* < 0.05 for all). Culture conditions and inter-specific interactions had interaction effects on the traits of both species. In monoculture treatments, the biomass production, height increment and side-shoot production of *S. angustifolium* was significantly higher in the laboratory than in the field (*p* < 0.01; Figure 1a–c). However, the biomass production of *S. magellanicum* was lower in the laboratory than in the field (*p* < 0.001; Figure 1a). As for the biochemical traits, the phosphorus content, reserved phenolics content, and released phenolics content of both species were lower in the laboratory than in the field (*p* < 0.05 for both; Figure 1g,h,k), while their carbon and nitrogen content showed the opposite results (*p* < 0.05 for both; Figure 1i,j and Table 1).

### 3.2. Phenolic Responses to the Neighbor

**Figure 2:**
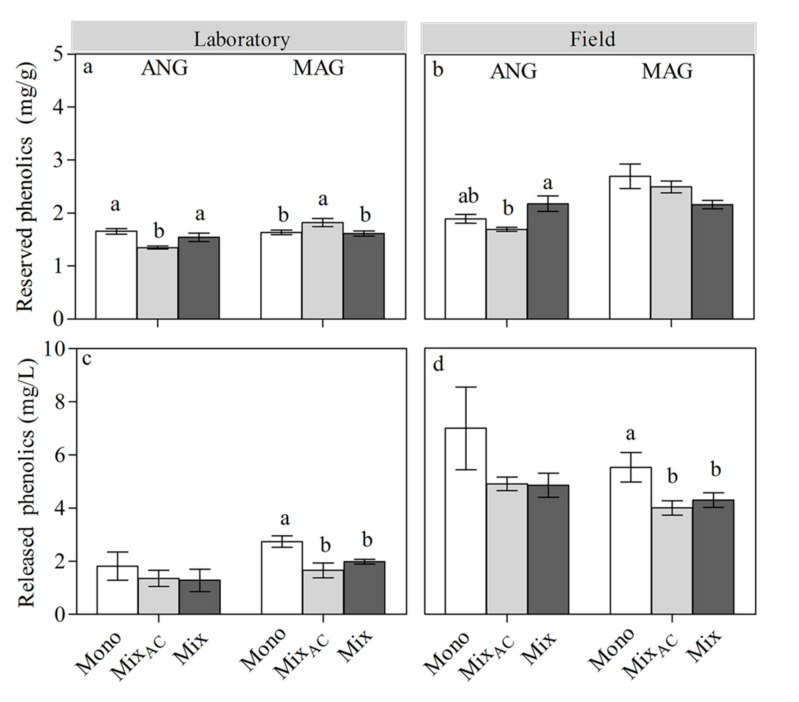
Effect of different inter-specific interactions on the shoot-reserved phenolics and released phenolics of ANG and MAG in the laboratory (**a**,**c**) and field (**b**,**d**). Data are mean ± 1SE (*n* = 5). The lowercase letters represent significant difference among different interaction.

In the laboratory, *S. angustifolium* did not show a response to the mixed culture without AC (activated charcoal) compared to the monoculture in reserved and released phenolics contents, but it showed a decrease of its reserved phenolics content in a mixed culture with AC (*p* = 0.014; Figure 2a,c). In the field, the reserved phenolics of *S. angustifolium* increased in the mixed culture without AC compared to the mixed culture with AC (*p* = 0.003; Figure 2b).

In *S. magellanicum*, compared to the monoculture, the responses of its reserved and released phenolics to the mixed culture without AC in the laboratory were the same as in the field. Its reserved phenolics content in mixed culture with AC was much higher than that of the monoculture (*p* < 0.01) and mixed culture without AC (*p* < 0.01), while its released phenolics content in a mixed culture with AC (*p* < 0.01) and a mixed culture without AC (*p* < 0.01) were lower than that of the monoculture (Figure 2b,d) in the laboratory.

### 3.3. Phenotypic Plasticity

**Figure 3:**
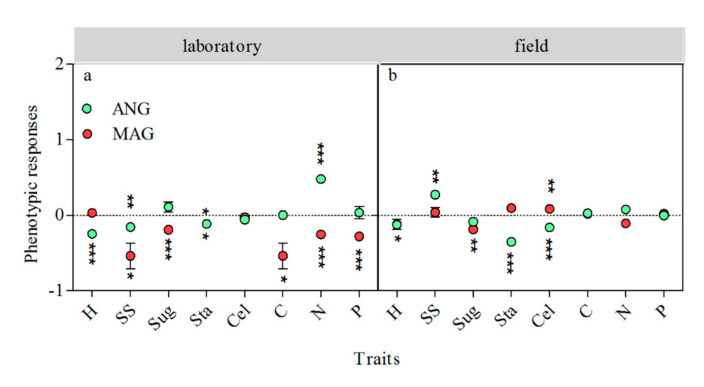
Effect of total inter-specific interactions on the phenotypic plasticity of *S. angustifolium* and *S. magellanicum* in the laboratory (**a**) and the field (**b**). The green and red dots represent the response amplitude of *S. angustifolium* and *S. magellanicum*, respectively. Data are mean ± 1SE (*n* = 5). Asterisks represent significantly difference from 0. *: *p* < 0.05; **: *p* < 0.01; ***: *p* < 0.001. The data of the biomass production, side-shoot production, reserved phenolics content, released phenolics content, soluble sugar content, and starch content of *Sphagnum* in the field were from Liu et al. (2020) [22].

In the laboratory experiment, the phenotypic plasticity responses of *S. magellanicum* to the total inter-specific interaction were mainly negative. Its side-shoot production (*p* < 0.05), soluble sugar content (*p* < 0.001), starch content (*p* < 0.05), carbon content (*p* < 0.05), nitrogen content (*p* < 0.001), and phosphorus content (*p* < 0.001) showed negative responses to the neighbor (Figure 3a). The effect of the total inter-specific interaction on the height increment (*p* < 0.001), side-shoot production (*p* < 0.01), and starch content (*p* < 0.05) of *S. angustifolium* were negative, and its nitrogen content (*p* < 0.001) had a positive response to the total inter-specific interaction (Figure 3a).

In the field experiment, the soluble sugar content (*p* < 0.01) of *S. magellanicum* showed a negative response to its neighbor, and cellulose content (*p* < 0.01) showed a positive response to its neighbor (Figure 3b). The side-shoot production (*p* < 0.01) of *S. angustifolium* showed a positive response to its neighbor, while its height increment (*p* < 0.05), starch content (*p* < 0.001), and cellulose content (*p* < 0.001) showed negative responses to the neighbor (Figure 3b).

### 3.4. Relative Interaction Intensity (RII)

**Figure 4:**
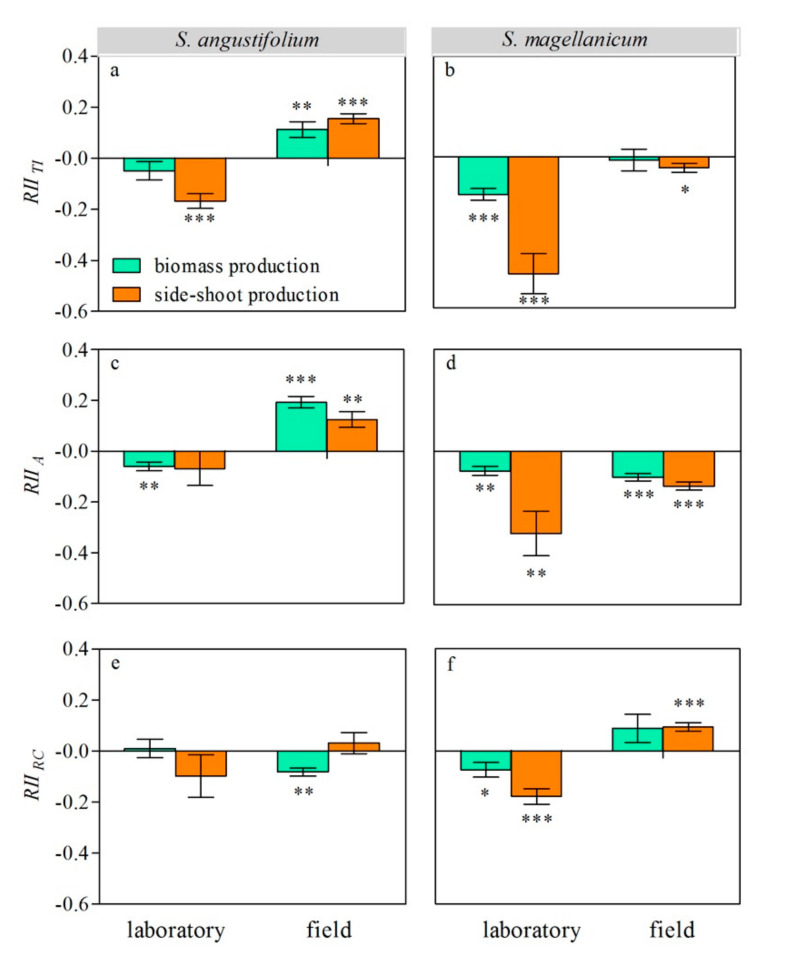
Relative interaction intensity (*RII*) of *S. angustifolium* (**a**,**c**,**e**) and *S. magellanicum* (**b**,**d**,**f**) under different inter-specific interactions (*TI*: total inter-specific interaction; *A*: allelopathy; and *RC*: competition) in the laboratory and the field. Data are mean ± 1SE (*n* = 5). Asterisks represent significantly difference from 0. *: *p* < 0.05; **: *p* < 0.01; ***: *p* < 0.001.

We used the relative interaction intensity (*RII*) index to indicate neighbor effects and interaction types. A positive value of *RII* indicated facilitation, and a negative value indicated inhibition. Both the relative total interaction intensity (*RII _TI_*) and relative allelopathy intensity (*RII _A_*) of the neighbor, *S. magellanicum*, on *S. angustifolium* were negative (*p* < 0.01 for both) in the laboratory, but they were positive (*p* < 0.01 for both) in the field (Figure 4a,c). *Sphagnum magellanicum* did not suppress *S. angustifolium* through resource competition in the laboratory but did so in the field (*RII _RC_* < 0 and *p* < 0.01; Figure 4e).

In the laboratory, *RII _TI_* and *RII _RC_* showed that *S. angustifolium* suppressed *S. magellanicum* (*p* < 0.01; Figure 4b,f). In the field, *RII _TI_* and *RII _C_* showed that the neighbor suppressed and promoted *S. magellanicum*, respectively (*p* < 0.05, Figure 4b,f). In both the laboratory and the field, *S. magellanicum* was suppressed by the allelopathic effect of its neighbor (*RII _A_* < 0 and *p* < 0.01 for both; Figure 4d).

**Figure 5:**
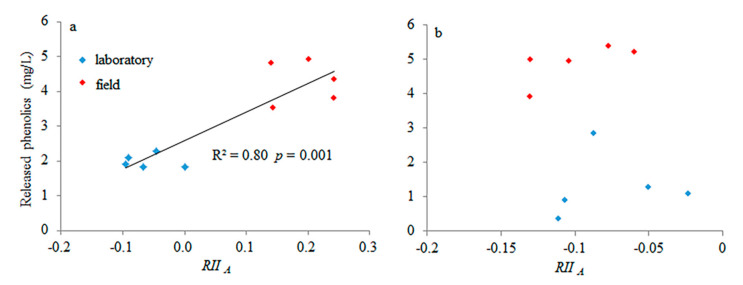
Relationship between the released phenolic concentration of the target species and the relative interaction intensity of allelopathy (*RII _A_*) based on the biomass production of the target species on the neighbor (**a**): *S. angustifolium* as the target species; (**b**): *S. magellanicum* as the target species. Blue and red diamonds represent the data measured in the laboratory and the field, respectively.

The more phenolics of *S. magellanicum* that were released, the more strongly it allelopathically affected *S. angustifolium* (Figure 5a). However, there was no relationship between the released phenolics of *S. angustifolium* and its relative allelopathic intensity (Figure 5b).

## 4. Discussion

### 4.1. Allelopathy and Resource Competition

The *RII* showed strong negative effects of *S. angustifolium* and *S. magellanicum* allelopathy on each other in the laboratory, which supported our first hypothesis. This is contrasted with the positive effects of *S. magellanicum* allelopathy on *S. angustifolium* in the field (negative relative neighbor effect found by Liu et al., 2020, and the negative *RII* calculated in the current study). In bryophytes, phenolics are considered to be the main category of allelochemicals [7] that may impose inhibitory or stimulatory effect on growth of microorganisms and seedlings or the germination of seeds and spores [16,37].

Generally, an allelopathic effect is content-dependent, with high allelochemical content showing a negative effect and a low allelochemical content showing positive effect [23]. However, contrary to such knowledge and the second hypothesis, a positive relationship between the released phenolics of *S. magellanicum* and its relative allelopathic intensity (Figure 5a) was observed, and no relationship was observed between the released phenolics of *S. angustifolium* and its relative allelopathic intensity, suggesting that the concentration of phenolics may not explain the type of allelopathy. This further means that inter-specific mechanisms except for released phenolics may result in positive effects of the allelopathy of *Sphagnum* in the field [22]. For instance, in the field, the strong drought stress in the non-rainy season may lead to facilitation among bryophytes by their inter-specific water conservation cooperation [20]. The water retention ability of hollow species is lower than that of hummock species [38]. Thus, such positive effects of allelopathy in the field may be attributed to the effects of the water supply provided from *S. magellanicum* on *S. angustifolium* instead of the positive effects of the phenolics from *S. magellanicum*. In addition, some researchers have suggested that overcompensation induced by plant defenses to herbivory is the most likely pathway for hormetic responses [39] that help them escape specific types of chemical stress [21].

Positive and negative effects of plants’ secondary metabolites are likely to occur simultaneously [40,41], and our experiment revealed the net allelopathic effects of *Sphagnum* on the growth of neighbors. Curiously, previous laboratory experiments have shown that *Sphagnum* mainly has no negative effect of the allelopathy on plants of the same genus. For example, the exudates of *Sphagnum palustre* increased the height increment of *S. magellanicum* [42], and the exudates of *S. magellanicum* promoted the biomass production of *S. wulfianum* [14]. Such results may be due to the species-specific allelopathy of *Sphagnum* causing positive or suppressive effects [14]. The effect of the phenolics from other species on *S. wulfianum* may be far less than that of its own. Another possibility might be that those studies used a different method from ours to assess the allelopathic effects of *Sphagnum*. In their experiments, the receptor plants were watered with exudates taken from a single *Sphagnum* species (e.g., Huneck et al. 1990 and Montenegro et al. 2009) [43,44] unlike the mixed culture in our study.

The resource competition of *Sphagnum* was more pronounced in the laboratory conditions than in the field conditions. We found that in the laboratory, the growth of *S. magellanicum* was inhibited by resource competition from *S. angustifolium*. In the 14-month field experiment, we did not observe such a result, which suggests that the ability of resource competition of *S. angustifolium* was only stronger than that of *S. magellanicum* in the laboratory conditions. It is likely that variable biotic and abiotic factors are not favorable for holding the competitiveness of *S. angustifolium* in field conditions. We observed that the monthly mean precipitation in July dropped by 30% compared with June, while the monthly mean temperature in July was the highest in the growing season (from May to October), which created a dryer condition in July. In drought conditions, the growth and competitive advantage of *S. angustifolium* would decrease because of its lower drought tolerance, while *S. magellanicum* could increase its photosynthetic capacity and grow well [38,45,46]. In the field, due to the greater growth and high drought tolerance of *S. magellanicum*, *S. angustifolium* did not occupy an overwhelming superiority. However, in contrast to the field environment, the continuously moist condition helped *S. angustifolium* to hold a competitive advantage, as indicated by both biomass and side-shoot production.

### 4.2. Phenotypic Responses to Total Inter-Specific Interaction

The response of plant morphology is usually caused by various metabolic activities. For instance, the changed carbon metabolism of plants would influence dry-matter accumulation. In this paper, the results also showed that the morphological responses of *S. angustifolium* and *S. magellanicum* to inter-specific interactions were in line with their carbon metabolism, and their side-shoot production and starch content both declined. The soluble sugar content of *S. magellanicum* also negatively responded to the neighbor. However, in the field, the consistent responses between growth and non-structural carbon (NSC) disappeared. The NSC of the two *Sphagnum* species showed a negative response to the neighbor, while their growth did not show the same response. In the field, it is likely that the total inter-specific interaction between the two species was not strong enough (Figure 4a,b) to affect growth except for influencing NSC accumulation.

We found that in the mixed culture in the laboratory, the nitrogen content in *S. angustifolium* increased. but it was decreased in *S. magellanicum*. This might have been related to the differential ability of the two species in acquiring nutrition. In peatland, hollow species can usually be called competitors with higher growth rates, while hummock species can be called tolerators with lower growth rates [11,31]. A competitor–tolerator (*S. magellanicum*) is more conservative at obtaining nutrients than the competitor (*S. angustifolium*). For example, like *S. angustifolium*, *Sphagnum fallax* is a strong competitor belonging to the same section (Cuspidata) that uptakes much more NH_4_^+^ and NO_3_^−^ than *S. magellanicum* [47]. However, in the field, no response of nitrogen content in the two *Sphagnum* species to the neighbor was observed presumably due to some ecological factors (such as drought) that might have played a role and obscured the interspecific effect on their nitrogen content. Similar studies have reported that as humidity decreased, the positive effect of *S. palustre* on the height increment of *Sphagnum capillifolium* disappeared [31]. Ge (2016) found that *Polytrichum strictum* decreased the nitrogen content of *S. palustre* only in dry conditions [42]. In the future, it is necessary to consider the interactive effects of neighbors with respect to abiotic factors.

In conclusion, regardless of laboratory or field conditions, the competitive advantage of *Sphagnum* is mediated by both resource competition and allelopathy, but the type and intensity of the inter-specific interactions are driven by environmental variability. Compared with the field experiment, the intensity of the interaction between *S. magellanicum* and *S. angustifolium* was stronger in the laboratory because of the elimination of variable environmental factors that may have obscured or even decreased the contribution of inter-specific interactions, especially allelopathy. However, the results obtained in the laboratory may not demonstrate the real ecological role of allelopathy in plant–plant interference in nature. An innovative experimental design and sophisticated analytical chemistry for plant-released allelochemicals are needed to understand contrasted effect of allelopathy under field and controlled laboratory conditions.

## Figures and Tables

**Table 1:** Two-way ANOVA for effects of conditions (laboratory and field, df = 1) and inter-specific interactions (denoted as ISI and including Mono (monoculture), Mix (mixed culture without activated charcoal added in the neighbor), and Mix_AC_ (mixed culture with activated charcoal added in the neighbor; df = 2) on the morphological traits and biochemical traits of the two *Sphagnum* species. Statistically significant values are in bold.

Source	*Sphagnum angustifolium*						*Sphagnum* *magellanicum*					
	Condition		ISI		Condition × ISI		Condition		ISI		Condition × ISI	
	*F*	*p*	*F*	*p*	*F*	*p*	*F*	*p*	*F*	*p*	*F*	*p*
B (g)	14.62	**0.001**	0.30	0.746	2.22	0.131	19.00	**0.001**	1.93	0.167	1.05	0.367
H (cm)	10.17	**0.004**	2.71	0.087	1.98	0.160	1.13	0.299	0.44	0.648	1.82	0.184
SS	23.18	**0.001**	1.22	0.314	2.27	0.125	2.14	0.157	5.80	**0.009**	6.09	**0.007**
Sug (mg g^−1^)	6.98	**0.014**	0.59	0.563	1.28	0.297	59.07	**0.001**	2.12	0.142	0.60	0.558
Sta (mg g^−1^)	81.05	**0.001**	10.97	**0.001**	1.67	0.209	86.41	**0.001**	1.12	0.342	0.86	0.434
Cel (mg g^−1^)	563.8	**0.001**	15.58	**0.001**	35.26	**0.001**	315.3	**0.001**	5.17	**0.014**	5.41	**0.011**
C (mg g^−1^)	108.1	**0.001**	2.30	0.122	5.89	**0.008**	342.3	**0.001**	6.70	**0.005**	8.90	**0.001**
N (mg g^−1^)	51.42	**0.001**	2.72	0.086	0.87	0.432	15.94	**0.001**	10.85	**0.001**	3.24	0.057
P (mg g^−1^)	200.3	**0.001**	0.62	0.548	0.96	0.396	94.98	**0.001**	1.43	0.260	1.13	0.339
ResP (mg g^−1^)	19.83	**0.001**	6.82	**0.005**	1.34	0.281	55.05	**0.001**	4.65	**0.020**	1.25	0.306
RelP (mg g^−1^)	46.60	**0.001**	2.11	0.143	0.81	0.458	52.23	**0.001**	4.47	**0.022**	0.88	0.430

B: Biomass production; H: height increment; SS: side-shoot production; Sug: soluble sugar; Sta: starch; Cel: cellulose; C: carbon content; N: nitrogen content; P: phosphorus content; ResP: reserved phenolics; and RelP: released phenolics.

## References

[1] Wardle D.A., Karban R., Callaway R.M. (2011). The ecosystem and evolutionary contexts of allelopathy. Trends Ecol. Evol..

[2] Rice E.L. (1983). Allelopathy.

[3] Whitehead J., Wittemann M., Cronberg N. (2018). Allelopathy in bryophytes—A review. Lindbergia.

[4] Clymo R.S., Gore A.J.P. (1983). Peat. Ecosystems of the World.

[5] Clymo R.S. (1984). The limits to peat bog growth. Philos. Trans. R. Soc. Lond. B Biol. Sci..

[6] Breemen N.V. (1995). How *Sphagnum* bogs down other plants. Trends Ecol. Evol..

[7] Verhoeven J.T.A., Liefveld W.M. (1997). The ecological significance of organochemical compounds in *Sphagnum*. Acta Bot. Neerl..

[8] Jassey V.E.J., Gilbert D., Binet P., Toussaint M.L., Chiapusio G. (2011). Effect of a temperature gradient on *Sphagnum fallax* and its associated living microbial communities: A study under controlled conditions. Can. J. Microbiol..

[9] Jassey V.E.J., Chiapusio G., Binet P., Buttler A., Laggoun-Défarge F., Delarue F., Bernard N., Mitchell E.A.D., Toussaint M.L., Francez A.J. (2013). Above-and belowground linkages in *Sphagnum* peatland: Climate warming affects plant-microbial interactions. Glob. Chang. Biol..

[10] Fenner N., Freeman C. (2011). Drought-induced carbon loss in peatlands. Nat. Geosci..

[11] Bu Z.J., Rydin H., Chen X. (2011). Direct and interaction-mediated effects of environmental changes on peatland bryophytes. Oecologia.

[12] Ma J.Z., Bu Z.J., Zheng X.X., Ge J.L., Wang S.Z. (2015). Shading enhances the competitive advantage of *Sphagnum fallax* in a simulation experiment. Mires Peat.

[13] Turetsky M.R., Bond-Lamberty B., Euskirchen E., Talbot J., Frolking S., Mcguire A.D., Tuittila E.S. (2012). The resilience and functional role of moss in boreal and arctic ecosystems. N. Phytol..

[14] Ingerpuu N., Vellak K. (2013). Growth depends on neighbors: Experiments with three *Sphagnum* L. species. J. Bryol..

[15] Bu Z.J., Sundberg S., Feng L., Li H.K., Zhao H.Y., Li H.C. (2017). The Methuselah of plant diaspores: *Sphagnum* spores can survive in nature for centuries. N. Phytol..

[16] Michel P., Burritt D.J., Lee W.G. (2011). Bryophytes display allelopathic interactions with tree species in native forest ecosystems. Oikos.

[17] Callaway R.M. (2007). Positive Interactions and Interdependence in Plant Communities.

[18] Rydin H. (1997). Competition among bryophytes. Adv. Bryol..

[19] Mulder C.P.H., Uliassi D.D., Doak D.F. (2001). Physical stress and diversity-productivity relationships: The role of positive interactions. Proc. Natl. Acad. Sci. USA.

[20] Okland R.H., Okland T. (1996). Population biology of the clonal moss *Hylocomium splendens* in Norwegian boreal spruce forests. II. effects of density. J. Ecol..

[21] Qin F.C., Liu S., Yu S.X. (2018). Effects of allelopathy and competition for water and nutrients on survival and growth of tree species in *Eucalyptus urophylla* plantations. For. Ecol. Manag..

[22] Liu C., Bu Z.J., Mallik A., Rochefort L., Hu X.F., Yu Z. (2020). Resource competition and allelopathy in two peat mosses: Implication for niche differentiation. Plant Soil.

[23] Rudolph H., Samland J. (1985). Occurrence and metabolism of sphagnum acid in the cell walls of bryophytes. Phytochemistry.

[24] Scasta J.D., Trostle C.L., Foster M.A. (2012). Evaluating Alfalfa (*Medicago sativa* L.) cultivars for salt tolerance using laboratory, greenhouse and field methods. J. Agric. Sci..

[25] Diamond J.M. (1983). Ecology: Laboratory, field and natural experiments. Nature.

[26] Bertness M.D., Callaway R.M. (1994). Positive interactions in communities. Trends Ecol. Evol..

[27] Johansson L. (1983). Effects of activated charcoal in anther cultures. Physiol. Plant..

[28] Mahall B.E., Callaway R.M. (1992). Root communication mechanisms and intracommunity distributions of two mojave desert shrubs. Ecology.

[29] Mensuali-Sodi A., Panizza M., Serra G., Tognoni F. (1993). Involvement of activated charcoal in the modulation of abiotic and biotic ethylene levels in tissue cultures. Sci. Hortic..

[30] Soudzilovskaia N.A., Graae B.J., Douma J.C., Grau O., Milbau A., Shevtsova A., Wolters L., Cornelissen J.H.C. (2011). How do bryophytes govern generative recruitment of vascular plants?. N. Phytol..

[31] Bu Z.J., Zheng X.X., Rydin H., Moore T., Ma J.Z. (2013). Facilitation vs. competition: Does interspecific interaction affect drought responses in *Sphagnum*?. Basic Appl. Ecol..

[32] Rudolph H., Kirchhoff M., Gliesmann S., Glime J.M. (1988). *Sphagnum* culture techniques. Methods in Bryology Nichinan: Hattori Botanical Laboratory.

[33] Singleton V.L., Rossi J.A. (1965). Colorimetry of total phenolics with phosphomolybdic-phosphotungstic acid reagents. Am. J. Enol. Vitic..

[34] Eshghi S., Tafazoli E., Dokhani S., Rahemi M., Emam Y. (2007). Changes in carbohydrate contents in shoot tips, leaves and roots of strawberry (*Fragaria x ananassa* Duch.) during flower-bud differentiation. Sci. Hortic..

[35] Armas C., Ordiales R., Pugnaire F.I. (2004). Measuring plant interactions: A new comparative index. Ecology.

[36] Valladares F., Sanchez-Gomez D., Zavala M.A. (2006). Quantitative estimation of phenotypic plasticity: Bridging the gap between the evolutionary concept and its ecological applications. J. Ecol..

[37] Feng L., Sebastian S., Mark O., Wu Y.-H., Wang M., Bu Z.J. (2018). Oxygen-deficiency and allelochemicals affect *Sphagnum* spore persistence in peatlands. Plant Soil.

[38] Rydin H. (1993). Interspecific competition between sphagnum mosses on a raised bog. Oikos.

[39] Belz R.G., Duke S.O. (2014). Herbicides and plant hormesis. Pest Manag. Sci..

[40] Stark S., Kytviita M.M., Neumann A.B. (2007). The phenolic compounds in *Cladonia* lichens are not antimicrobial in soils. Oecologia.

[41] Pizňak M., Bačkor M. (2019). Lichens affect boreal forest ecology and plant metabolism. S. Afr. J. Bot..

[42] Ge J. (2016). Competiiton and Allelopathy Among Three Bryophytes in Hani Peatland of the Changbai Mountains.

[43] Huneck S., Meinunger L. (1990). Plant Growth Regulatory Acitivities of Bryophytes, a Contribution to the Chemical Ecology of Mosses and Liverworths.

[44] Montenegro G., Portaluppi M.C., Salas F.A., Díaz M.F. (2009). Biological properties of the Chilean native moss *Sphagnum magellanicum*. Biol. Res..

[45] Glime J.M. (2006). Bryophytes and herbivory. Cryptogamie Bryol..

[46] Granath G., Strengbom J., Rydin H. (2010). Rapid ecosystem shifts in peatlands: Linking plant physiology and succession. Ecology.

[47] Jauhiainen J., Wallén B., Malmer N. (1998). Potential NH_4_^+^ and NO_3_^−^ uptake in seven *Sphagnum* species. N. Phytol..

